# Acute angle closure attack after an intravitreal bevacizumab injection for branch retinal vein occlusion: a case report

**DOI:** 10.1186/s12886-017-0417-3

**Published:** 2017-03-14

**Authors:** Seongyong Jeong, Min Sagong, Woohyok Chang

**Affiliations:** 1 Department of Ophthalmology, Yeungnam University College of Medicine, 170, Hyunchungro, Nam-gu, Daegu, 42415 South Korea; 2 Chang’s Retina Center, 151, Jungang-daero, Nam-gu, Daegu, 42417 South Korea

**Keywords:** Intravitreal injection, Bevacizumab, Acute angle closure, Complication, Intraocular pressure

## Abstract

**Background:**

Intravitreal injection is widely used to treat retinal vein occlusion, and acute angle closure (AAC) is an exceptional complication of intravitreal injection. The authors report a case of AAC that occurred immediately after administering intravitreal bevacizumab to treat branch retinal vein occlusion (BRVO).

**Case presentation:**

A 65-year-old woman was referred to the retina clinic of a tertiary referral center for the treatment of macular edema secondary to BRVO. On slit lamp examination, anterior chamber (AC) depth was shallow (3 corneal thicknesses centrally, 1/4 corneal thicknesses peripherally) in both eyes. Intraocular pressure (IOP) was 19 mmHg in both eyes, and refractive error was +1.00 diopter sphere in both eyes. A gonioscopy exam demonstrated narrow angle of over 180° in both eyes. To treat the macular edema, bevacizumab was injected into her right eye intravitreally. After two bevacizumab injections, the macular edema regressed but recurred 5 months later, and thus, a third injection was performed. The next day, she visited our emergency department complaining of persistent ocular pain in her right eye. The right pupil had dilated to 6 mm diameter and was fixed. Slit lamp exam revealed diffuse corneal edema in her right eye, which had an IOP of 56 mmHg. After administration of intravenous mannitol, the IOP fell to 14 mmHg and the corneal edema disappeared. Subsequently, a glaucoma specialist performed laser iridotomy on the right eye.

**Conclusions:**

Although AAC is a rare complication of intravitreal injection, it can occur in a patient with risk factors such as hyperopic eye or narrow angle.

## Background

Intravitreal injections are extensively used to treat various retinal diseases, and bevacizumab, a humanized monoclonal anti-vascular endothelial growth factor (VEGF) antibody is a common therapeutic agent for exudative age-related macular degeneration and macular edema resulting from diabetic retinopathy or retinal vein occlusion (RVO). Because of its proven efficacy and relatively low-cost, the adoption of intravitreal bevacizumab injection is increasing in retinal clinics, and therefore, the identification of its adverse effects is important.

Intravitreal injection has been reported to have several complications, such as, intraocular inflammation, vitreous hemorrhage, retinal detachment, endophthalmitis and intraocular pressure (IOP) elevation [1]. Because of the volume effect in the closed intravitreal cavity, IOP elevation appears inevitable after an intravitreal injection. However, many authors agree IOP spikes after intravitreal injection are transient and that additional intervention, such as anterior chamber (AC) paracentesis is not required [2–4]. Nevertheless, IOP elevation may induce morphologic change of AC angle which may worsen outflow of aqueous humor. Here, we present a case of acute angle closure (AAC) onset after intravitreal bevacizumab injection in an eye with macular edema resulting from branch retinal vein occlusion (BRVO). Only one case report of AAC after intravitreal injection in a patient with central retinal vein occlusion has been previously published [5], and to our knowledge, no case of AAC after intravitreal injection in patient with BRVO has been reported.

## Case presentation

**Fig. 1:**
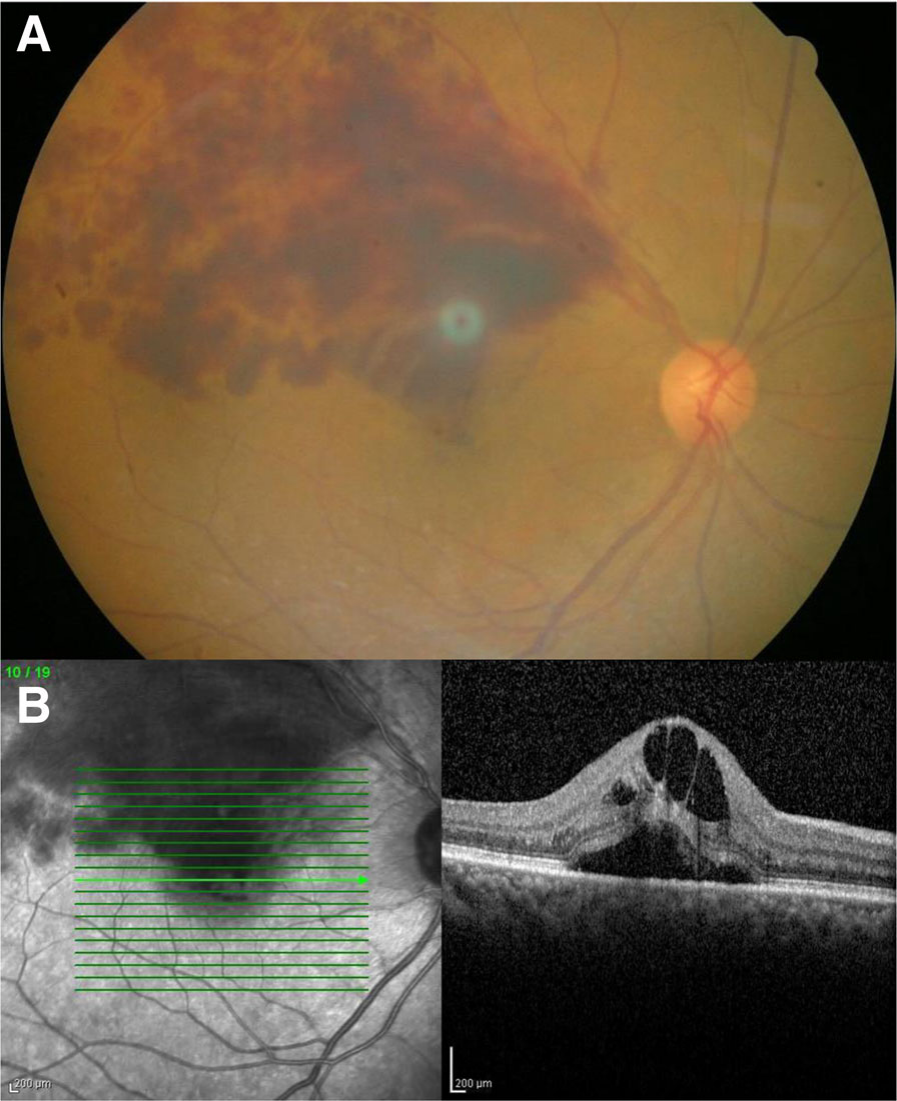
Fundus photography (**a**) and OCT (**b**) of right eye; **a** Fundus photography showing extensive flame-shaped hemorrhage along the superotemporal retinal vein. **b** Optical coherence tomography showing cystoid macular edema and subretinal fluid

A 65-year-old woman was referred to the retina clinic of tertiary referral center with decreased vision of 3 days duration in her right eye. She had no remarkable past medical or family history. Her best corrected visual acuity was 0.15 in the right eye and 1.0 in the left. IOP was 19 mmHg in both eyes. There was no afferent pupillary defect of right or left pupils. On slit-lamp examination, sclera and conjunctiva showed no injection, and there was no corneal edema in either eye. AC was relatively shallow (3 corneal thicknesses centrally, 1/4 corneal thicknesses peripherally) in both eyes, and no inflammation was observed in either eye. Lenses showed mild nuclear cataract in both eyes. A gonioscopy exam demonstrated a narrow angle of over 180° in both eyes. The refractive error was +1.00 diopter sphere in each eye. Dilated fundus exam revealed extensive flame-shaped hemorrhage along the superotemporal vein and macular edema in the right eye (Fig. 1). The optic nerve head appeared normal with no evidence of glaucomatous excavation and a cup-to-disc ratio of 0.5 in both eyes. The remaining fundus examination was unremarkable in the left eye.

Optical coherence tomography (OCT) depicted intraretinal multiple cystic spaces and subretinal fluid (SRF) around fovea in the right eye (Fig. 1). Central retinal thickness (CRT) was 677 μm in the right eye. Fluorescein angiography of the right eye revealed a delayed filling time of the involved superior retinal vein.

Intravitreal bevacizumab (1.25 mg/0.05 mL) was injected into the right eye using a 30 gauge needle. The injection site was pressed for 10 s with a cotton-tipped applicator to prevent bevacizumab reflux. One month after this injection, the cystoid macular edema had almost regressed and CRT was 256 μm. Visual acuity of the right eye had increased to 0.5. To resolve the remaining edema and SRF, second intravitreal bevacizumab injection was administered to the right eye, and 1 month after this second injection, macular edema and SRF had regressed, CRT was 236 μm, and visual acuity was 0.6.

Five months after the second bevacizumab injection, macular edema recurred and OCT showed intraretinal multiple cystic spaces with a CRT of 479 μm. Visual acuity was 0.5 in right eye. We immediately injected an intravitreal bevacizumab a third time into the right eye. All injections were performed by one retinal specialist using the same method. After injetion, notable complications were not observed, and the patient did not complain any symptoms at that time. Gross visual acuity was routinely checked after injection and she was able to count fingers.

**Fig. 2:**
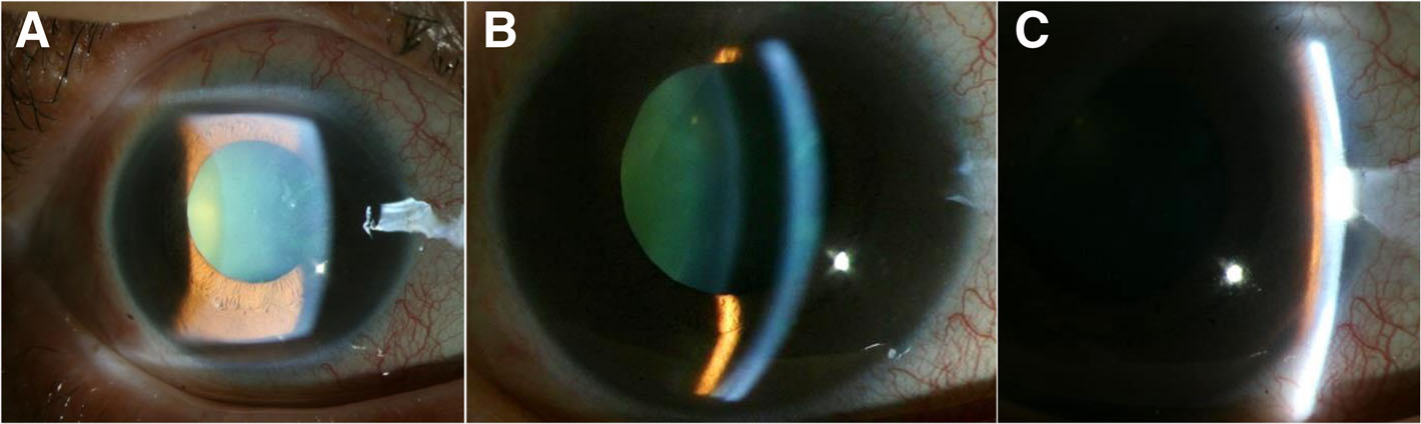
Anterior segment photography of the right eye; **a** The pupil was mid-dilated and fixed. Corneal edema and punctate leison were observed. **b** Central anterior chamber depth was about 3 corneal thicknesses. **c** Peripheral chamber depth was about 1/4 corneal thickness

The next day, the patient presented to our emergency department complaining of persistent ocular pain. The patient now mentioned that this pain had started after the intravitreal injection. She also complained of headache, nausea, and vomiting. At this presentation, visual acuity was 0.08 in the right eye and 0.9 in the left. The left pupil was normal but the right pupil was fixed and mid-dilated (6 mm). A slit-lamp exam revealed diffuse epithelial edema of cornea in the right eye. AC depth was similar to that observed at her first visit (3 corneal thicknesses centrally, 1/4 corneal thicknesses peripherally) in both eyes (Fig. 2). IOP was 56 mmHg in the right eye and 15 mmHg in the left. A diagnosis of acute angle-closure glaucoma was made and she was immediately treated with 300 ml of 20% mannitol intravenously. One hour after mannitolization, IOPs in right and left eyes were 14 and 16 mmHg, respectively, and epithelial edema of the right eye had decreased. Finally, a glaucoma specialist performed laser iridotomy (LI) to the right eye. After 3 days, prophylactic LI to the left eye was performed to prevent the potential risk of AAC.

One month after LI, IOPs in right and left eyes were 13 and 15 mmHg, respectively, and iridotomy sites were patent in both eyes. Visual acuity was 0.7 in the right eye and 1.0 in the left. Automated visual field testing was performed using the Humphrey 750i (Carl Zeiss Meditec, Dublin, California, USA) with the 24–2 Swedish interactive threshold algorithm (SITA) standard program and no glaucomatous visual field defect was evident in either eye. On dilated fundus examination, the optic nerve showed no glaucomatous change in either eye. Previous macular edema and flame-shaped hemorrhage had almost disappeared in the right eye, and OCT revealed normal peripapillary retinal nerve fiber layer thickness in both eyes and no macular edema in the right eye. Ocular biometry was performed using IOL master (Carl Zeiss Meditec, Dublin, California, USA). In right and left eyes, AC depths were 2.42 and 2.12 mm, respectively, and axial lengths were 22.64 and 22.50 mm, respectively.

## Discussion and conclusions

This is likely the first case report to be issued on acute angle closure after an intravitreal injection in branch retinal vein occlusion.

Although the safety of intravitreal injection with respect to IOP elevation has been established [2, 3], some aspects require careful consideration. In previous studies, most patients tolerated intravitreal injections with IOP normalization within 30 min but a few cases reached an IOP of ~80 mmHg immediately after injection [3, 6]. Because of these exceptional cases with extreme IOP elevation, further studies are needed to identify high risk patients and caution should be taken when a patient is considered at risk.

The extreme IOP elevation can be explained by biomechanical model, in which IOP elevation after intravitreal injection may depend on the ocular biometric characteristics of treated eye [7, 8]. According to this theory, hyperopic eyes with short axial length and a small intraocular volume are at greater risk of extreme IOP elevation after intravitreal injection because they may possess stiffer sclera rigidity, and a greater percentage of intraocular volume is introduced than in myopic eyes with long axial length. Benz et al. reported an association between immediate IOP changes and vitreous reflux after intravitreal triamcinolone acetonide injections [6], and noted eyes without vitreous reflux showed greater IOP elevations than eyes with reflux. In addition, eyes with preexisting glaucoma took significantly longer after intravitreal injection to achieve an IOP of < 30 mmHg [3].

In our case, the patient had a hyperopic eye of short axial length and IOP normalization probably took longer because of the presence of an outflow pathway abnormality (narrow angle). Furthermore, the operator prevented vitreous reflux by blocking the injected site using a cotton-tipped applicator, which would have contributed to IOP elevation.

Increased IOP may induce morphologic changes in the AC because of forward movement of the iris and lens. Alkin el al. reported an AC depth decrease (measured by anterior segment OCT) 5 min after an intravitreal injection when the IOP was significantly higher than baseline [9]. However, with time, the IOP normalized to baseline and AC depth normalized and correlated with IOP level. We presume anterior displacement of the anterior segment structure may have altered angle configuration and result in further obstruction of aqueous humor outflow pathway in our patient, and that this could have led to an AAC attack followed by further rapid IOP elevation.

The mydriasis before intravitreal injection may also contribute to the development of AAC attack. It has been suggested in previous studies that mydriatic agents may precipitate AAC attack in narrow angle patients [10].

Intravitreal bevacizumab injection is commonly used to treat various retinal diseases but special care should be taken when it is used to treat macular edema resulting from RVO for two reasons. The first is RVO may be associated with narrow angle [11]. In a retrospective case series study of 19 patients with RVO and narrow angle, it was suggested angle closure should be considered as a risk factor of RVO [12]. The authors recommended angle status be checked when patients present with RVO. The second reason is that despite the rarity of AAC and the exceptional occurrence of AAC after intravitreal injection [5], AAC is important because it can cause irreversible optic nerve damage leading to visual field loss and blindness [13]. Therefore, an evaluation of angle configuration should be taken before intravitreal injection, especially in the patients with RVO. If risk factors such as narrow angle were identified, detailed investigating symptoms or IOP check as well as gross visual acuity assessment may be needed after intravitreal injection.

In summary, IOP elevation after intravitreal injection is usually non-hazardous. However, special caution is needed in patients with predisposing factors such as hyperopic eyes or narrow angle. Furthermore, because of the possible association between narrow angle and RVO, the angle status should be checked before administering an intravitreal injection in RVO patients.

## Abbreviations

AAC: Acute angle closure
AC: Anterior chamber
BRVO: Branch retinal vein occlusion
IOP: Intra ocular pressure
LI: Laser iridotomy
OCT: Optical coherence tomography
RVO: Retinal vein occlusion
SITA: Swedish interactive threshold algorithm
VEGF: Vascular endothelial growth factor
